# Emergency Department Utilization Among Undocumented Latino Patients During the COVID-19 Pandemic

**DOI:** 10.1007/s40615-022-01382-8

**Published:** 2022-08-18

**Authors:** Annie Ro, Tim A. Bruckner, Michael Pham Huynh, Senxi Du, Andrew Young

**Affiliations:** 1 Department of Health, Society, and Behavior, Anteater Instruction and Research Building (AIRB), UC Irvine Program in Public Health, 653 E. Peltason Road, Irvine, CA 92697 USA; 2 Keck School of Medicine, University of Southern California, Los Angeles, CA USA; 3 Division of Geriatric, Hospital, Palliative and General Internal Medicine, Department of Medicine, Keck School of Medicine, Los Angeles, CA USA

**Keywords:** Emergency service, COVID-19, Undocumented immigrants, Hispanic Americans, Time series analysis

## Abstract

**Objective:**

To determine whether Latino undocumented immigrants had a steeper decline in Emergency Department (ED) utilization compared to Latino Medi-Cal patients in a Los Angeles safety-net hospital, March 13, 2020, to May 8, 2020.

**Study Design:**

The data were extracted from patient medical records for ED visits at LAC + USC Medical Center from January 2018 to September 2020. We analyzed weekly ED encounters among undocumented Latino patients in the nine-week period after COVID was declared a national emergency. We applied time-series routines to identify and remove autocorrelation in ED encounters before examining its relation with the COVID-19 pandemic. We included Latino patients 18 years of age and older who were either on restricted or full-scope Medi-Cal (*n* = 230,195).

**Results:**

All low-income Latino patients, regardless of immigration status, experienced a significant decline in ED utilization during the first nine weeks of the pandemic. Undocumented patients, however, experienced an even steeper decline. ED visits for this group fall below expected levels between March 13, 2020, and May 8, 2020 (coef. =  − 38.67; 95% CI =  − 71.71, − 5.63). When applied to the weekly mean of ED visits, this translates to a 10% reduction below expected levels in ED visits during this time period.

**Conclusion:**

Undocumented immigrants’ health care utilization was influenced by external events that occurred early in the pandemic, such as strict stay-at-home orders and the public charge rule change. Health care institutions and local policy efforts could work to ensure that hospitals are safer spaces for undocumented immigrants to receive care without immigration concerns.

## Introduction

The COVID-19 pandemic drastically changed patterns of healthcare utilization in the USA. In particular, emergency department (ED) visits plummeted during the earliest days of the pandemic, as stay-at-home orders proliferated and patients avoided seeking health care for non-COVID-19 related conditions. Hospital surveillance data indicated a 42% decline in ED visits nationwide in March and April 2020 compared to the same period in 2019, a mean of 2.1 million fewer visits per week [1]. Other data from specific states and hospital systems also found substantial declines [2–4].

One group that might have experienced large declines in ED use during the early COVID-19 pandemic is undocumented immigrants. There are an estimated 10.5 million undocumented immigrants in the USA [5] and 2.7 million in California, the setting of this study [6]. Undocumented immigrants were already disconnected from the health care system before the COVID-19 pandemic. Nearly 90% of low-income undocumented immigrants in California are uninsured [7] and have a limited availability of health services. During the COVID-19 pandemic, federal initiatives such as the Coronavirus Aid, Relief, and Economic Security (CARES) Act and Families First Coronavirus Response Act (FFCRA) did not offer financial relief to undocumented immigrants, exacerbating their resource-related barriers to accessing care [8].

The early stages of the pandemic also coincided with the public charge rule change instituted by the Trump Administration at the end of February 2020. The new rule expanded the definition of a “public charge” to include a broader set of public resources that could bar immigrants from receiving a green card, opening the potential for undocumented immigrants to respond by avoiding health care visits [8]. In addition, Immigration and Customs Enforcement (ICE) never formally banned immigration enforcement activities at medical centers during the pandemic, contributing further to the perception of the hospital as an unsafe place. Other work has shown that undocumented immigrants avoid seeking health care when immigration enforcement is high [9]. Despite their heightened sensitivity to the earliest days of the COVID-19 pandemic, we know almost nothing about undocumented immigrants’ health care utilization during this time.

There is little information on the patterning of ED utilization during the early part of the COVID-19 pandemic by social factors. The few available studies suggest that racial/ethnic minority and low-income patients, which includes undocumented immigrants, avoided the ED at greater levels than did non-Latino White and higher socioeconomic status (SES) patients [10–12]. For example, in a California hospital system, Latino patients experienced a 6% decline in ED visits between March 2019 and March 2020 compared to a 6% increase among non-Latino White patients. Patients who lived in high-poverty neighborhoods and had Medi-Cal (the California equivalent of federal Medicaid) also experienced steeper declines in ED use compared to patients in low-poverty neighborhoods and covered by commercial care, respectively [10]. The uneven decline in ED use by race and ethnicity and socioeconomic status has implications on health disparities, as low SES and racial/ethnic minority patients are already at higher risk for certain chronic conditions. In data from an Alabama hospital system, Latino and African American patients appeared to be foregoing needed medical attention, as they showed a larger drop in high acuity ED visits than non-Latino White patients and for a longer period of time [12]. Delayed or foregone care can create more severe health problems in the long run [13], compounding the already-high disease burden among racial/ethnic minority and low-income patients.

This study aims to examine changes in ED utilization for Latino undocumented immigrants versus Medi-Cal patients in the largest safety net hospital in Los Angeles (LA) County, California, in the earliest days of the pandemic. We focused on LA County for two reasons. First, LA is the second largest health system of any county in the USA. Its size yields generally stable measures over time of ED visits—an important consideration for our analytic procedures. Second, the county has nearly 880,000 undocumented immigrants, which is the largest county-level population of undocumented immigrants in the country. This not only provides a large enough sample size to detect a range of effects, but can also provide insight into population trends among the undocumented immigrant population in the state.

This was an especially sensitive time for undocumented immigrants, as it included the most restrictive stay-at-home orders as well as the threat of the public charge rule change. We also make methodological improvements over past research that has examined declines in ED visits during the early days of the pandemic. Most of these studies use a “stacked calendar” approach that compares ED visits in 2019 to the same time period in 2020 and calculates percent change between the two time points. In contrast, we use data from well before the pandemic to account for broader patterns, including trend and seasonal variations in ED visits, to determine whether COVID-19, as a plausibly external “shock”, created ED utilization patterns that deviate from expected patterns. We expect that, because of immigration-related barriers, undocumented low-income Latino immigrants experienced steeper declines in ED utilization during COVID-19 pandemic when compared to documented low-income Latino patients. This paper also addresses an important gap in ED research by documenting potential disparities in ED utilization between documented and undocumented immigrants.

## Methods

### Data

We analyzed all ED visits to the Los Angeles County + University of Southern California (LAC + USC) Medical Center between January 5, 2018, and September 24, 2020. For reasons described below, we divided the period into 142 7-day periods of ED visit data. The 408,928 records were “treat and release” outpatient ED visits; we conducted sensitivity checks that included ED visits that resulted in an inpatient stay. We limited our sample to visits among self-reported Latino patients 18 years of age and older who were either insured by restricted Medi-Cal or full-scope Medi-Cal, leaving us with a sample of 230,195 ED encounters over the 2.5-year period. Patients report their ethnicity (Latino/Non-Latino) at intake, which is recorded by a provider. All data were de-identified to conform to Health Insurance Portability and Accountability Act (HIPAA) requirements.

### Variables

#### Immigration Status

We used primary payer status of the encounter to approximate immigration status and compared undocumented patients to full-scope Medi-Cal patients. We coded a patient as having undocumented status if restricted-scope Medi-Cal was the primary payer source for the ED encounter. In California, restricted-scope Medi-Cal, or “Emergency Medi-Cal,” provides health coverage for emergency services for patients who meet the income threshold for Medi-Cal but do not meet immigration status requirements as either US nationals, citizens, or lawful permanent residents [14]. Previous work has also used this measure as a proxy for undocumented status [15, 16]. We chose full-scope Medi-Cal (hereafter referred to as Medi-Cal) patients as a comparison group of low-income patients who are either US-born or foreign-born with authorized status. Because of the program’s “lawful status” requirements, we can assume, consistent with past literature that all of the Medi-Cal patients are either citizens or lawfully residing in the USA.

#### COVID-19 Stay-at-Home Orders

We aggregated ED visits, by race and ethnicity and health insurance type, to 7-day periods. We summed ED visits starting on Fridays and ending on Thursdays, so that Friday, March 13, 2020, the date that the Trump Administration declared a national emergency due to COVID-19, serves as the first day of the exposed “anchor” 7-day period. On Monday, March 16, 2020, moreover, LA County ordered closure of public schools, bars, gyms, entertainment centers, and indoor dining. This circumstance led us to examine 142 full weeks of ED visits beginning Friday, January 5, 2018, and ending Thursday, September 24, 2020. These 7-day periods, which we refer to as weeks for simplicity, represent the longest time series available to us at the time of our test. The key independent variable is a binary variable for the 1^st^ societal shutdown in LA County, coded as 1 for the nine weeks from March 13 to May 8, 2020, and coded as 0 for all other weeks.

#### ED Visits

We used the count of ED visits among undocumented Latino patients by week as the dependent variable. All project activities were reviewed and approved by the University of Southern California Institutional Review Board (HS-19–00,890), which served as a reliance for the University of California, Irvine Institutional Review Board.

### Analysis

Our approach applies time-series methods which are increasingly used to examine the influence of “interruptions” such as COVID-19 [17–19]. ED visits among undocumented Latino patients may exhibit patterns including seasonality, trend, and the tendency for high or low values to be “remembered” into subsequent months (see Bruckner et al., 2012 for an example of auto-regressive “memory” of ED visits LA County). These patterns, referred to as autocorrelation, complicate tests of association because the expected value of a patterned series is not its mean.

As recommended by statisticians and time-series researchers [21, 22], we address this autocorrelation problem in two ways. First, we use as a control series the count of ED visits among Latino patients on *Medi-Cal* in that same week. The Latino Medi-Cal control series absorbs any patterns in ED visits shared by both undocumented and Medi-Cal enrolled Latino patients that may arise from, for example, cultural shifts in help-seeking, changes in population size, or the well-documented downward trend in all medical visits during COVID-19 societal restrictions. Second, we employ autoregressive, integrated, moving average routines (ARIMA), devised by Box and Jenkins, to identify and remove any remaining autocorrelation in the dependent variable series. These routines express autocorrelation as a product of “autoregressive” (AR), “integrated” (I), and “moving average” (MA) parameters, collectively referred to as ARIMA models. The residuals of these ARIMA models meet the assumptions of correlational tests in that they have an expected value of 0 and exhibit no serial dependence.

We implemented the above time-series approach with the following steps. First, for the ED visits among undocumented Latino patients, we used Box and Jenkins transfer function modeling to express their weekly counts in week *t* as a function of ED visits among Medi-Cal-insured Latinos patients in week *t* [22]. Second, we added ARIMA parameters to the transfer function to express autocorrelation identified in its residual values (i.e., error term). Third, we estimated the test model formed by adding the first stay-at-home orders restriction binary variable into the model resulting from step 2. We specified a synchronous relation such that we hypothesize a fall in ED visits among undocumented Latino patients during the first stay-at-home orders. We used a two-tailed test. Fourth, we inspected the residuals of the time-series equation to ensure that they exhibited no autocorrelation. Fifth, we assessed the stability of results to outliers. Outliers in the time series could artificially inflate standard errors and lead to a type II error (false acceptance of the null). We therefore used outlier detection and correction routines as recommended in the time-series literature [23]. This method iteratively adds binary variables for each week to find any that, if added to the equation, would have coefficients with *t* values greater than 3.5. The method also adjusts the ARIMA parameters as outliers are added. We used software from Scientific Computing Associates for all steps (version 5.4.6, SCA Corp., Villa Park, IL).

## Results

During the test period from January 5, 2018, to September 24, 2020, there were a total of 161,693 ED visits from our analytic sample. Most patient encounters were Medi-Cal (52.2%), female (55.2%), and of younger ages 18–34 (25.9%) (Table 1). A diagnosis for other lower respiratory disease was one of the top six reasons for an ED visit in 2020 but not in 2018 or 2019.

**Table 1:** ED visits to LAC + USC Medical Center 2018–2020, self-reported Latino patients 18 years of age and older who were either on restricted Medi-Cal or full-scope Medi-Cal

	2018 (*n* = 62,239)		2019 (*n* = 64,740)		2020 (*n* = 34,714)		Total (*n* = 161,693)	
	*N*	%	*N*	%	*N*	%	*N*	%
Proportion of all visits Latino		69.86		71.33		68.97		70.24
Insurance status								
Undocumented	30,126	48.40	31,177	48.16	15,980	46.03	77,283	47.80
Medicaid	32,113	51.60	33,563	51.84	18,734	53.97	84,410	52.20
Sex								
Female	34,845	55.99	36,075	55.72	18,336	52.82	89,256	55.20
Male	27,377	43.99	28,644	44.24	16,358	47.12	72,379	44.76
Unknown	17	0.03	21	0.03	20	0.06	58	0.04
Age								
18–34	16,331	26.24	16,452	25.41	9,032	26.02	41,815	25.86
35–44	12,684	20.38	13,267	20.49	7,079	20.39	33,030	20.43
45–55	14,539	23.36	14,941	23.08	8,105	23.35	37,585	23.24
55–64	11,092	17.82	11,732	18.12	6,371	18.35	29,195	18.06
65 +	7,593	12.20	8,348	12.89	4,127	11.89	20,068	12.41
Top reasons for visit								
Abdominal pain	4,425	7.11	5,028	7.77	2,665	7.68	12,118	7.49
Other connective tissue disease	2,142	3.44	2,414	3.73	1,213	3.49	5,769	3.57
Nonspecific chest pain	2,088	3.35	2,286	3.53	1,140	3.28	5,514	3.41
Spondylosis; intervertebral disc disorders; other back problems	2,102	3.38	2,263	3.50	1,069	3.08	5,434	3.36
Other non-traumatic joint disorders	2,123	3.41	2,341	3.62	956	2.75	5,420	3.35
Headache; including migraine	2,033	3.27	2,265	3.50	979	2.82	5,277	3.26

**Fig. 1:**
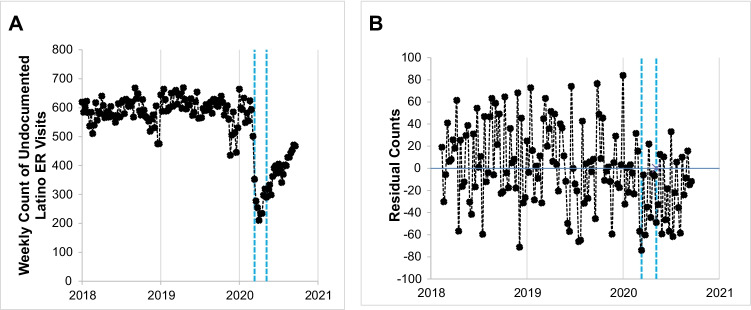
Count of ED visits among undocumented Latino patients over 142 weeks spanning January 5, 2018, to September 24, 2020, in Los Angeles County, CA. Legend: Panel A plots the observed count; Panel B plots the residual count, with mean = 0, after controlling for ED visits among Medi-Cal-insured Latino patients and removal of autocorrelation (first 6 weeks lost to time-series modeling). The first stay-at-home period (March 13 to May 8, 2020) is indicated with double dotted vertical lines. The first week in January of each year is demarcated with a single vertical line. (Note diff)

Over the test period, the mean number of weekly ED visits among undocumented Latino patients was 544.25 (*SD* = 107.19; range: 210 to 669). This value is slightly less than that among the control population of Latino Medi-Cal-insured patients (mean = 571.08, *SD* = 91.14, range: 253 to 719). The plot of ED visits among undocumented Latino patients (Fig. 1, Panel A) shows substantial week-to-week variation, and its mean before March 2020 appears relatively stable. Beginning in March 2020, however, ED visits declined substantially, which coheres with other reports of COVID-19-related declines in help-seeking more broadly.

Figure 1, Panel B plots the results of the first two time-series steps in which we specified a transfer function that (i) controlled for shared patterning between the dependent variable series and that of the “control” Latino Medi-Cal-insured ED visit series, and (ii) controlled for any discovered residual autocorrelation. The first 6 weeks of the plot were lost to ARIMA modeling. Box-Jenkins routines detected AR(1) and AR(5) parameters such that high (or low) values in ED visits were followed by similarly high (or low) values 1 and 5 weeks later, albeit in diminishing amounts. The “5-week” memory, we speculate, may capture calendar month effects when using weekly data (i.e., memory of “first of the week” effects from month-to-month). The residuals plotted in Fig. 1, Panel B have a mean of 0 and their weekly values are serially independent of one another. Visual inspection of the eight weeks beginning March 13, 2020 (dotted line), the date the Trump Administration declared a National Emergency due to COVID-19, suggests that the residual values of ED visits appear lower than 0.

Table 2 shows the results from our final test equation for ED visits among undocumented Latino patients. ED visits for this group fell below expected levels during the first stay-at-home orders (coef. =  − 38.67; 95% CI: − 71.71, − 5.63). We then applied outlier detection and control routines to determine whether unusually large or small weekly counts of ED visits reduced the efficiency of our estimates by inflating confidence intervals. We detected two outliers: one temporary change at Week 81 (July 19, 2019 to July 25, 2019), and a level shift at Week 124 (May 15, 2020 to May 21, 2020). The outlier at Week 124—the week that the first societal restrictions were lifted—likely reflects the sustained drop in ED help-seeking during COVID-19, even after the first stay-at-home orders. Adjustment for these outliers shrank confidence intervals and increased the magnitude of the point estimate of the first stay-at-home orders by 40% (coef. =  − 54.33; 95% CI: − 78.57, − 30.09).

**Table 2:** Time series results predicting the count of emergency department visits among undocumented Latino patients in Los Angeles County during the first COVID-19 stay-at-home orders as a function of Latino Medi-Cal patients and autocorrelation, from January 5, 2018 to September 24, 2020 (standard errors in parentheses)

Parameter	Initial model		Outlier-adjusted model	
	Coefficient	95% CI	Coefficient	95% CI
Latino Medi-Cal patients	0.96	(0.94, 0.97)	0.97	(0.96, 0.98)
AR(1)	0.28	(0.12, 0.44)	0.09	(− 0.08, 0.25)
AR(5)	0.19	(0.03, 0.36)	0.06	(− 0.10, 0.23)
1st stay-at-home orders	− 38.67	(− 71.71, − 5.63)	− 54.33	(− 78.57, − 30.09)

To give the reader a sense of the magnitude of our main result, we estimated from the outlier-adjusted model the number of ED visits statistically “avoided” by the first stay-at-home orders. Multiplication of the outlier-adjusted first stay-at-home orders coefficient by its 8-week duration yielded approximately 435 fewer ED visits than expected among undocumented Latino patients. When applied to the weekly mean of ED visits, the first stay-at-home orders coincided with a 9.98% reduction below expected levels in ED visits over this time period. We note that this result may represent a lower bound of the reduction given that we controlled for the general COVID-19-related drop in ED visits shared across the Medi-Cal-insured and undocumented Latino populations.

Although our hypothesis pertains to ED visits during to the first stay-at-home orders, the plot in Fig. 1, Panel B compelled us to explore the potential change in ED visits among undocumented Latino patients during the entire COVID-19 pandemic period. We therefore repeated our time-series steps but now specified the independent variable as a COVID-19 binary switch, coded as “1” for all weeks March 13, 2020, and thereafter, and “0” otherwise. Our results, consistent with those from the original test, supported a sustained drop in ED visits (coef: − 47.59; 95% CI: − 63.77, − 31.41).

The sharp decline in ED visits during March 2020, even among our control population of Medi-Cal-insured Latino patients, led us to explore whether Latino patients within the Medi-Cal group reduced help-seeking during the first societal shutdown relative to non-Latino Medi-Cal patients. We explored this possibility by repeating the time-series steps as described in the methods but now used Latino Medi-Cal patients as the dependent variable and non-Latino Medi-Cal patients as a control series. Results within Medi-Cal, consistent with previous literature, indicated a reduction in ED visits among Latino relative to non-Latino patients, during the first societal shutdown (Supplemental Table 1; coef: − 92.08; 95% CI: − 147.83, − 36.32).

### Sensitivity Check

Our primary analysis sample was among treat-and-release ED visits. As a sensitivity check, we included ED visits that resulted in a hospital stay to determine the robustness of our results inclusive of more acute ED visits (*n* = 259,153). The results consistently indicated a significantly lower number of ED visits among undocumented immigrants during the first stay-at-home orders (Supplemental Table 2 coef. =  − 49.92; 95% CI =  − 86.10, − 13.72). The size of the coefficients increased slightly, owing to the overall higher number of ED visits when inpatient admissions are included.

## Discussion

We examined ED utilization among undocumented Latino immigrants during the earliest stages of the COVID-19 pandemic in the largest safety net hospital in Los Angeles County. We found that all low-income Latino patients, regardless of immigration status, experienced a significant decline in ED utilization when the first stay-at-home orders were issued in LA county. Undocumented patients, however, experienced an even steeper decline than Medi-Cal-insured Latino patients. They saw a 10% drop in ED utilization compared to expected levels, which translates to 435 fewer ED visits for undocumented immigrants during this period. The drop in ED use among undocumented immigrants seemed to extend even beyond the initial nine-week stay-at-home orders, suggesting that this prolonged decline lasted at least through the fall of 2020.

There has been little research to date on how undocumented immigrants fared during the early days of the COVID-19 pandemic, despite multiple theoretical commentaries that have identified undocumented status as a major health care barrier during the pandemic [8, 24]. Our results confirm that undocumented immigrants were highly vulnerable to ED avoidance during the first stay-at-home orders. While other research has found that Latino patients show a greater decline in ED use relative to non-Latino Whites during the early stages of the pandemic [10], we found an even steeper decline among undocumented Latino immigrants *compared to other low-income Latinos*. This means the undocumented population significantly curtailed their ED utilization even more than the already-lower ED visits among Latino patients generally.

The decline in ED visits we observed among Latinos generally may have been attributable to a greater perceived threat of COVID or higher barriers to care during the stay-at-home order found among racial/ethnic minority and low SES patients. Other work has found that racial and ethnic minorities reported a higher fear of COVID-19 and viewed it as a major threat to the population, even in the earliest days of the pandemic [25]. This greater perceived risk could have deterred Latino patients from going to the ED and exposing themselves to COVID-19 infection. Additionally, stay-at-home orders could have differentially reduced available financial resources for racial/ethnic minority and low-income patients to seek care at the ED. For example, Latinos experienced elevated levels of unemployment, more food insecurity, and lower total spending throughout the pandemic [26].

In addition to reduced ED access during the stay-at-home order found among racial and ethnic minorities generally, undocumented immigrants’ health care utilization is also sensitive to immigration-specific concerns. The results of our study align with others that have found Latino undocumented immigrants’ health care utilization to be affected by immigration threats [27]. The start of the pandemic coincided with the start of the new public charge rule change. The combination of the stay-at-home orders and the added threat of punitive immigration policy could have had a multiplicative effect on ED avoidance among undocumented patients. The downturn occurred despite a state policy that expanded restricted Medi-Cal (i.e., the ED payment source for most uninsured undocumented immigrants) to cover COVID-19 testing and treatment at no cost to the patient [28]. This suggests that expanding access may not improve undocumented immigrants’ health care utilization if there are other concerns about immigration threats.

Future work could consider the long-term health implications of foregoing ED visits on undocumented immigrants’ health status. While undocumented immigrants generally use the ED less than other Latinos and tend to have better physical health [29, 30], the ED remains one of their main sources of healthcare [31]. The ED is likely the first point of contact for many undocumented immigrants, making it imperative that patients know it is a secure place to receive medical care without fear of immigration enforcement. In qualitative research, undocumented immigrants made connections between COVID-19 hospitalization and immigration enforcement, believing COVID-19 infection and associated care to be a surreptitious way to uncover immigration status [32]. One possible approach to encourage necessary medical care is through community health workers, who are crucial resources to help undocumented patients overcome barriers to health care access related to their immigration status [33].

Our findings should be considered in light of some methodological limitations. We did not have a direct measure of immigration status and used insurance payment as a proxy measure. We feel reasonably confident in this approach, as other studies in peer-reviewed literature have used this method as well [16]. Patients who are not insured at the time of the ED encounter but are Medi-Cal eligible are coded under a separate payment source that provides qualified individuals immediate access to temporary Medi-Cal while applying for permanent Medi-Cal or other health coverage (e.g., Hospital Presumptive Eligibility, HPE). We also acknowledge that our data is limited to the first six months of the pandemic and may not capture longer-term trends. The ARIMA method is a population-level analysis and does not control for individual characteristics. We use Latino Medi-Cal patients as a control group, which accounts for shared factors that could influence ED use as whole, such as changes in policy or data collection artifacts. The interrupted time series design also uses the undocumented patient population as its own control, comparing the ED visits from the same population before and after the stay at home orders. Our results could be biased by individual covariates if there was a drastic change during the stay-at-home orders only (i.e., age, language, marital status), and only in undocumented (but not Medi-Cal patients), but we do not believe this joint set of circumstances is likely. Another limitation is that we did not consider subgroup differences among Latino immigrants, who may have different patterns of utilization [34]. Yet the ethnic composition between undocumented and Medi-Cal patients is likely similar, as Mexicans, Salvadorians, and Guatemalans make up the majority of both the general Latino population in Los Angeles County as well as the undocumented population of the county [35, 36]. Lastly, our results may not be generalizable to other regions of the USA. Undocumented immigrants in Los Angeles may be more familiar with and have more trust in the local healthcare system due to local county programs that offer health care resources to the Los Angeles undocumented population. Thus, this study may underestimate the reduction in ED utilization among undocumented patients.

Undocumented immigrants’ health care utilization is precarious and affected by external events. Health care institutions and local policy efforts could work to ensure that hospitals are safe spaces for undocumented immigrants to receive care without concerns over their immigration status. If ED avoidance continues as the COVID-19 pandemic evolves, local groups and care organizations could expand outreach efforts to undocumented populations to ensure their continuity of care in other settings, such as outpatient care.
